# FIB-4, APRI, and ALRI as Predictors of COVID-19 Outcomes: Insights from a Large-Scale Study

**DOI:** 10.3390/diagnostics15161984

**Published:** 2025-08-08

**Authors:** Anita Aminzadeh, Nazanin Azmi-Naei, Maryam Teimouri, Marzieh Rohani-Rasaf

**Affiliations:** 1 Student Research Committee, School of Public Health, Shahroud University of Medical Sciences, Shahroud 3614773955, Iran; anita.aminzadeh@gmail.com; 2 Department of Epidemiology, School of Public Health, Shahroud University of Medical Sciences, Shahroud 3614773955, Iran; nazaninazmi1375@gmail.com; 3 Department of Clinical Biochemistry, School of Allied Medical Sciences, Shahroud University of Medical Sciences, Shahroud 3614773955, Iran; m.teimouri20@gmail.com; 4 Center for Health Related Social and Behavioral Sciences Research, Shahroud University of Medical Sciences, Shahroud 3614773955, Iran

**Keywords:** COVID-19, FIB-4, APRI, ALRI, inflammatory indices, prognosis, severity

## Abstract

**Background:** Simple and cost-effective biochemical markers are still very useful for predicting severity and mortality in COVID-19 patients. This study investigates the association of some inflammatory and also non-invasive biochemical indices of liver function and critical care outcomes of COVID-19 patients. **Methods:** In this cross-sectional study, a total of 2232 hospitalized COVID-19 patients, regardless of the presence of underlying liver diseases, were followed. Based on the laboratory results at the time of admission, five indices—FIB-4 (Fibrosis-4), NLR (Neutrophil to Lymphocyte Ratio), APRI (Aspartate Aminotransferase to Platelet Ratio), ALRI (Aspartate Aminotransferase to Lymphocyte Ratio), and SII (Systemic Immune-Inflammation)—were calculated. **Results:** According to the results of multivariate regression, all five indices were predictors of mortality and severity in COVID-19 patients after adjusting for age, sex, comorbidities and BMI. The odds ratios for FIB-4, NLR, APRI, ALRI, and SII to predict mortality were 1.14 (1.07–1.21), 1.07 (1.04–1.1), 1.28 (1.12–1.46), 2.44 (1.76–3.38), and 1.57 (1.13–2.17), respectively. For predicting severity, the odds ratios were 1.22 (1.15–1.30), 1.09 (1.06–1.11), 1.78 (1.44–2.21), 1.73 (1.41–2.14), and 1.27 (1.04–1.57), respectively. Additionally, based on the AUC results, FIB-4 and NLR indices demonstrated the best performance in predicting COVID-19 mortality and severity, respectively. **Conclusions:** Our results show that the non-invasive biochemical indices of liver function, NLR, and SII can be useful as early predictors of severity and mortality in COVID-19 patients.

## 1. Introduction

Despite widespread vaccination programs, coronavirus disease (COVID-19) is still considered as an acute respiratory syndrome with highly potent pathogenicity and transmissibility [1]. The clinical manifestations of the disease are diverse, from asymptomatic to the severe form, highlighting the importance of identifying patients with a high risk of the severe form of disease [2].

Approximately 5% of COVID-19 patients develop the advanced and severe form of the disease [3]. Studies have shown that most cases of acute respiratory distress syndrome (ARDS) are older patients or individuals with comorbid conditions, particularly diabetes, hypertension, cancers, liver diseases, chronic kidney diseases and others [3]. Specifically, regarding liver diseases, evidence suggests that while coronavirus itself can lead to liver damage, pre-existing liver abnormalities are considered significant risk factors linked to the severity and mortality of COVID-19 [4]. A systematic review and meta-analysis of 88 studies involving 6,653,207 COVID-19 cases indicated that liver disease is associated with increased hospitalization and mortality, even after adjusting for age and gender [5]. Hence, identifying simple and cost-effective biochemical indices associated with liver function can be useful for the prediction of severity and mortality in COVID-19 patients and the improvement of medical care and services to COVID-19 patients [6].

Although some studies have used liver transaminases to define liver damage [7] and have shown that individuals with more severe COVID-19 have higher levels of transaminases, the use of these biochemical markers has some limitations. For example, the elevation of these enzymes can also occur in other conditions, such as muscle injuries, and on the other hand, liver patients may not show changes in transaminase levels [8].

The FIB-4 (Fibrosis-4) index is a cost-effective and non-invasive index that is calculated using commonly available laboratory results, including platelet count, aspartate aminotransferase (AST), alanine aminotransferase (ALT), and age, to identify liver fibrosis [9]. It has high prognostic value for liver-related outcomes, such as hepatocellular carcinoma, severe fibrosis, and cirrhosis [4]. This index also helps detect liver damage in patients with nearly normal or only slightly elevated liver transaminase levels, potentially reducing the number of unnecessary biopsies required to identify liver damage [10,11].

Additionally, research has shown that this index is also associated with mortality in COVID-19 patients, even in the absence of pre-existing liver disease [12,13]. Furthermore, indices such as the Aspartate Aminotransferase to Platelet Ratio Index (APRI) and the Aspartate Aminotransferase to Lymphocyte Ratio Index (ALRI), which are also related to liver damage, appear to be potential predictors of disease severity in COVID-19 [14,15].

However, most studies in this area have examined the prognostic effects of these indices for a single outcome, such as the need for mechanical ventilation [16,17], or have focused on specific populations of COVID-19 patients with underlying conditions [18,19]. Moreover, no comprehensive comparison of the performance of liver injury indices for predicting COVID-19 severity and mortality has been conducted in Iran. Therefore, this study aims to evaluate the predictive capabilities of the FIB-4, APRI, and ALRI indices, as well as inflammatory markers such as the Neutrophil to Lymphocyte Ratio (NLR) and Systemic Immune-Inflammation Index (SII), for severity and mortality in all COVID-19 patients, regardless of the presence or absence of pre-existing liver disease.

## 2. Materials and Methods

### 2.1. Study Design and Participant

In this cross-sectional observational study, we evaluated a total of 2232 eligible patients. The inclusion criteria for this study included hospitalized and non-vaccinated cases with a COVID-19 diagnosis confirmed by real-time reverse transcription-polymerase chain reaction (RT-PCR) for oro- and nasopharyngeal swab specimens from the beginning of the COVID-19 pandemic until early March 2021, and those who had visited Imam Hossein Hospital at Shahroud University (located in northeastern Iran) under the supervision of Shahroud University of Medical Sciences. Based on the exclusion criteria, COVID-19 patients who did not undergo the necessary tests to determine the FIB-4, NLR, APRI, ALRI, and SII indices, as well as individuals who did not consent to the use of their medical records, were excluded from the study.

This study was approved by the Ethics Committee of the Research Unit of Shahroud University of Medical Sciences (IR.SHMU.REC.1401.200), and informed consent was obtained from all participants before data collection.

### 2.2. Non-Invasive Assessment of Liver Function

All necessary laboratory parameters required to calculate the useful indices, including APRI, FIB-4, ALRI, NLR, and SII, were measured from patients’ blood samples within 24 h of admission, after 10 h fasting, and applied to the following formulas. Finally, based on the scores obtained from these equations and considering the cut-off values derived from the ROC curve with the highest sensitivity and specificity in the studied population, the optimal points and the area under the curve (AUC) for all these indices were determined to identify the best prognostic value (see Section 2.5).

FIB-4 = [age × AST (IU/L)]/[platelets (×10^9^) × √ALT (IU/L)]

ALRI = AST value (U/L)/lymphocyte count (×10^9^/L)

APRI = [100/Platelet count (×10^9^/L)] × [AST value (U/L)/upper limit normal]

SII = Platelet count × Neutrophil count/Lymphocyte count (×10^9^/L)

NLR = Absolute Neutrophil count/Absolute Lymphocyte count

### 2.3. Outcome

The primary outcomes and dependent variables examined in this study are as follows: 1. Mortality in COVID-19 patients. 2. Disease severity among the participants. Disease severity is defined based on the following variables: respiratory rate > 30 breaths/min, oxygen saturation < 93%, mortality cases, ICU admission, and the need for mechanical ventilation.

### 2.4. Another Covariate

The patients’ demographic characteristics (age and gender), body mass index (BMI), inflammatory marker C-reactive protein, and history of all comorbidities based on self-reports by patients or their companions at the time of admission (including various cancers, history of chemotherapy, diabetes, AIDS, cardiovascular disease, asthma, chronic obstructive pulmonary disease (COPD), chronic liver and kidney diseases, seizures and neuro-cerebral diseases, immune system-related diseases such as lupus, rheumatoid arthritis, and MS) were included in the model under two groups: with/without underlying conditions.

### 2.5. Statistical Analysis

Data are reported as mean and standard deviation (SD) or median and interquartile range (IQR) for continuous variables, and as frequencies and percentages for categorical variables. The χ^2^ test was used to determine associations for qualitative and categorical variables, while the *t*-test was applied for continuous variables (*p* value < 0.05). Independent variables, along with potential confounders, were entered into a multivariate logistic regression model, where confounders were adjusted. Based on this, the odds ratios for each marker in predicting the severity and mortality of COVID-19 patients were calculated and compared (SPSS 23). Additionally, as a secondary objective, we determined the optimal cut-off point for each marker’s effect on the severity and mortality of COVID-19 by plotting ROC curves, calculating sensitivity and specificity at all points, determining the Youden index (the point where the sum of sensitivity and specificity is highest), and comparing the area under the curve (AUC).

## 3. Results

The baseline characteristics of 2232 patients are presented in Table 1. The mean age was 58.94 ± 16.75 years, with males comprising 50.2% of the patient population. Additionally, the mean BMI of the participants was 27.76 ± 4.60, classifying them as overweight, with a significant difference observed among the survivor and deceased groups (*p* = 0.006). Over half of the patients (53%) had comorbidities (53%). Furthermore, only 1.5% of the patients were diagnosed with liver disease.

There was a significant difference in the value of white blood cells (WBCs), red blood cells (RBCs), and hemoglobin (Hb) among deceased and severe individuals compared to survivors and non-severe patients (*p* < 0.001). In terms of hematocrit (HCT), there was no significant difference between the survivor and deceased groups (*p* < 0.001), while a significant difference was observed between the non-severe and severe groups (*p* = 0.123). The mean ALT level was 36.05 ± 46.39, and the mean AST level was 39.61 ± 44.12. While AST levels were significantly different between severe and non-severe patients (*p* < 0.001), ALT levels did not show any significant difference (*p* = 0.873; Table 2). The mean ALP was 183.34 ± 100.49, showing a significant difference between the survivor and deceased groups, as well as the non-severe and severe groups. Furthermore, the mean FIB-4 and APRI values were 2.38 ± 2.33 and 0.58 ± 1.08, respectively. The deceased group had a significantly higher mean FIB-4 of 4.08 ± 4.36 compared to the survivor group, which had a mean FIB-4 of 2.2 ± 1.91 (*p* < 0.001). Similarly, severe patients reported a higher mean FIB-4 (3.36 ± 3.61) compared to non-severe patients (mean FIB = 2.02 ± 1.48) (*p* < 0.001). The same pattern was observed with ALRI, NLR, and SII values, which were significantly higher in the severe and deceased groups compared to the non-severe and survivor patients (Table 2).

Multivariate logistic regression results indicated that for every 1-unit increase in FIB-4 and NLR, the odds of severity increased by 22% and 9% times, respectively. The analysis also demonstrated a similar relationship between these indices and mortality (see Table 3). Additionally, the odds of severity and mortality in COVID-19 patients were 1.27 and 1.57 times higher, respectively, in the high-SII group (>580) compared to the low-SII group (<580) after controlling for other variables in the model. The area under the curve (AUC) was employed to predict severity and mortality. FIB-4 showed better predictive ability for severity, while NLR was a better predictor for mortality compared to other markers.

For mortality, FIB-4 had a cut-off value of 2.19, with a sensitivity of 70% and specificity of 64%. For severity, a sensitivity of 65% and specificity of 60% corresponded to the FIB-4’s cut-off value of 1.96, marking it as an effective prognostic marker. ALRI had the lowest AUC for both severity and mortality (0.561 and 0.619, respectively) (Table 4).

## 4. Discussion

The use of simple and accessible biomarkers for the early detection and monitoring of COVID-19 patients, and the transferring of them to the ICU in severe cases, is of great importance. A good biomarker should have characteristics of simplicity in understanding, interpretation, and use, and ease of access. For this purpose, identifying biomarkers with these features can be highly effective in the early detection of disease severity in COVID-19 patients. Our study investigated the association between liver-associated and inflammatory indices and a poor prognosis in COVID-19.

Based on the laboratory findings at the admission time, we found that the FIB-4, ALRI, APRI, NLR, and SII indices were significantly associated with the risk of poor prognosis, including severity and mortality, in COVID-19 patients, regardless of the presence of underlying liver diseases.

Based on the initial findings of this study, although the levels of AST and ALT in deceased people were higher than survivors, only the AST level was significantly higher in the severe group compared to the non-severe group. This result is consistent with a systematic review and meta-analysis which found that AST levels were higher compared to ALT in severe COVID-19 patients [20]. It has also been suggested that studies generally show higher AST levels compared to ALT in severe COVID-19 patients [21]. In the present study, the AST/ALT ratio was also higher in the severe and deceased groups compared to the mild and surviving groups, respectively.

To date, AST/ALT ratios have been predominantly used as markers for assessing the severity of liver disease and as predictive factors for cirrhosis. A ratio of less than 1 indicates mild liver injury, while a ratio greater than 1 suggests severe hepatocyte damage [22]. Therefore, based on the findings of the present study, the increase in the AST/ALT ratio among the severe or deceased patients is likely due to greater liver damage. However, it is important to note that AST, unlike ALT, is not a specific marker for diagnosing liver dysfunction. Elevated levels of AST in severe COVID-19 patients could be attributed to other factors, such as cardiac or muscular damage or an immune response [23].

The significantly higher FIB-4 index in the severe and deceased groups compared to the non-severe and surviving groups may indicate the relevance and importance of this index in determining the status of COVID-19 patients in the current study. This finding is consistent with the results of a cohort study involving 992 COVID-19 patients, which found that an increase in FIB-4 was associated with higher mortality and disease severity [21]. According to the results of this study, patients with FIB-4 scores greater than 3.25 showed a higher prevalence of severe respiratory failure and pneumonia, confirming FIB-4 as a significant risk factor for the progression of the disease, even after adjusting for other influencing variables [7]. In the present study on COVID-19 patients, a FIB-4 score greater than 2.19 predicted mortality, while a score above 1.96 predicted disease severity. As shown in Table 3 and through regression model analysis, the suitability of this index as a tool for predicting severity and mortality has been demonstrated, aligning with previous studies. For example, studies by Adina Kamal [24] and Tommaso Bucci [7] introduced FIB-4 as a simple tool for predicting the status of patients at a high risk of severe COVID-19 infection, highlighting its superiority over traditional methods for diagnosing liver damage.

Although APRI showed a significant association with the severity and mortality of COVID-19 patients in the current study, findings from various studies on this topic have been inconsistent. For instance, in a study by Yijia Li and colleagues, the predictive ability of APRI for mortality was not statistically significant. This might be due to the small sample size in the mentioned study (*n* = 202), while the current study, with a larger sample size, was able to provide more reliable results [8]. On the other hand, several studies have supported our finding of a significant association between APRI and critical care outcomes [25,26,27]. Moreover, consistent with the present study, Reyes-Ruiz et al. showed that ALRI levels were significantly higher among the deceased patients, with ALRI > 42.42 identified as a risk factor for mortality in COVID-19 patients [28]. Also, numerous studies have demonstrated that while both FIB-4 and APRI levels are elevated in deceased patients, only FIB-4 has shown a statistically significant association with mortality in COVID-19 patients, exhibiting higher predictive performance [7,13,29].

Regarding inflammatory indices, in this study, the NLR index was significantly associated with both disease severity and mortality. In line with our study results, Liu et al. [30] and Fu et al. [31] reported that NLR could be an appropriate predictor of critical care outcomes in COVID-19 patients. Another marker examined in this study was SII, which was shown to be a risk factor for mortality in COVID-19 patients, as demonstrated in a study by Ayşegül et al. [14]. Furthermore, according to a systematic review and meta-analysis conducted in 2023, SII levels were significantly higher in patients with more severe outcomes and in deceased individuals compared to those with milder outcomes and survivors [32].

This research is a comprehensive study with a large sample size that was performed to explore the correlation between some inflammatory and also non-invasive biochemical indices of liver function and critical care outcomes of COVID-19 patients. This study also determines the prognostic utility of these indices in COVID-19 patients in Iran.

The study’s limitations include the restriction to only patients’ laboratory results from the time of hospital admission, with laboratory data gathered prior to COVID-19 infection not being accessed. Additionally, it was not possible to include non-hospitalized patients. Furthermore, there was a lack of phenotypic and histological data to identify liver disorders, such as fatty liver disease. Moreover, the cross-sectional nature of the study design limits the ability to confirm the temporal and causal relationship between exposure and outcome. Repeating and confirming this study through a prospective analysis would certainly strengthen the findings and enhance their generalizability.

## 5. Conclusions

In summary, our results showed that the three non-invasive biochemical indices of liver function (FIB-4, APRI, and ALRI) and two inflammatory indices (NLR, and SII) can be useful as early predictors of severity and mortality in COVID-19 patients. Also, the findings of this study suggest that the use of these five simple and cost-effective indices for the monitoring and medical care of COVID-19 patients would be beneficial. Our finding also highlight the high risk of critical care outcomes among COVID-19 cases with liver dysfunction.

## Figures and Tables

**Table 1:** The background features of participants with COVID-19 stratified by the severity and mortality.

Variable		Total (*n* = 2232)	Survivor (*n* = 2018)	Deceased (*n* = 214)	*p*-Value	Non-Severe (*n* = 1639)	Severe (*n* = 593)	*p*-Value
Age		58.94 ± 16.75	57.65 ± 16.50	71.15 ± 14.05	<0.001	56.19 ± 16.18	66.53 ± 15.97	<0.001
Sex, *n* (%)	Female	1111 (49.8)	1021 (50.6)	90 (42.1)	0.18	843 (51.4)	268 (45.2)	0.009
	Male	1121 (50.2)	997 (49.4)	124 (57.9)		796 (48.6)	325 (54.8)	
BMI, kg/m^2^		27.76 ± 4.60	27.85 ± 4.58	26.89 ± 4.79	0.006	27.78 ± 4.44	27.71 ± 5.05	0.780
Comorbidity (%)		1183 (53)	1032 (51.1)	151 (70.6)	<0.001	794 (48.4)	389 (65.6)	<0.001
Liver disease		34 (1.5)	32 (1.6)	2 (0.9)	0.460	25 (1.5)	9 (1.5)	0.990

Data are expressed as mean ± standard deviation of mean or number (percent).

**Table 2:** Laboratory findings of patients with COVID-19 stratified by severity and mortality.

Variable		Total (*n* = 2232)	Survivor (*n* = 2018)	Deceased (*n* = 214)	*p*-Value	Non-Severe (*n* = 1639)	Severe (*n* = 593)	*p*-Value
WBCs (×10^9^/L)		3.69 ± 2.44	3.49 ± 2.11	5.61 ± 3.96	<0.001	3.29 ± 1.74	4.80 ± 3.50	<0.001
RBCs (×10^6^/µL)		4.63 ± 0.69	4.66 ± 0.64	4.48 ± 0.7	<0.001	4.7 ± 0.6	4.5 ± 0.74	<0.001
Hb (g/dL)		13.16 ± 1.9	13.2 ± 1.87	12.80 ± 2.09	0.007	13.29 ± 1.81	12.75 ± 2.1	<0.001
HCT (%)		39.80 ± 4.83	39.86 ± 4.75	39.32 ± 5.52	0.123	40.03 ± 4.51	39.19 ± 5.58	<0.001
ALT (IU/L)		36.05 ± 46.39	34.69 ± 37.32	48.86 ± 95.72	<0.001	34.67 ± 30.84	39.87 ± 73.87	0.873
AST (IU/L)		39.61 ± 44.12	37.35 ± 37.1	60.94 ± 82.76	<0.001	35.72 ± 25.1	50.35 ± 73.72	<0.001
ALP (IU/L)		183.34 ± 100.49	180.22 ± 97.44	212.51 ± 121.94	<0.001	178.58 ± 99.21	196.68 ± 102.90	<0.001
CRP (%)	Positive	1920 (86)	1735 (90.1)	185 (89.4)	0.746	1406 (89.6)	514 (91.3)	0.237
	Negative	213 (9.5)	191 (9.9)	22 (10.6)		164 (10.4)	49 (8.7)	
FIB-4		2.38 ± 2.33	2.2 ± 1.91	4.08 ± 4.36	<0.001	2.02 ± 1.48	3.36 ± 3.61	<0.001
APRI		0.58 ± 1.08	0.54 ± 0.77	1.01 ± 2.51	<0.001	0.50 ± 0.44	0.81 ± 1.94	<0.001
NLR		4.39 ± 4.63	4.09 ± 4.25	7.19 ± 6.71	<0.001	3.81 ± 3.39	5.97 ± 6.75	<0.001
ALRI		69.94 ± 89	66.31 ± 81.95	104.08 ± 134.29	<0.001	64.78 ± 81.18	84.14 ± 106.42	<0.001
SII		939.27 ± 1239.76	870.7 ± 1095.79	1585.97 ± 2065.06	<0.001	811.40 ± 913.90	1291.33 ± 1817.90	<0.001

Abbreviations: WBCs = white blood cells, RBCs = red blood cells, Hb = hemoglobin, HCT = hematocrit, ALT = alanine transferase, AST = aspartate transferase, ALP = alkaline phosphatase, CRP = C-reactive protein.

**Table 3:** Estimating the predictive ability of some indices for mortality and severity in COVID-19 patients using logistic regression.

	Mortality			Severity		
	OR (CI)	*p*-Value	AUC	OR (CI)	*p*-Value	AUC
FIB-4	1.14 (1.07–1.21)	<0.001	0.763	1.22 (1.15–1.30)	<0.001	0.712
APRI	1.28 (1.12–1.46)	<0.001	0.761	1.78 (1.44–2.21)	<0.001	0.708
NLR	1.07 (1.04–1.1)	<0.001	0.770	1.09 (1.06–1.11)	<0.001	0.709
ALRI (low-ALRI group as reference) *	2.44 (1.76–3.38)	<0.001	0.768	1.73 (1.41–2.14)	<0.001	0.702
SII (low-SII group as reference) **	1.57 (1.13–2.17)	<0.001	0.756	1.27 (1.04–1.57)	<0.001	0.696

Adjusted for age, sex, BMI, comorbidity. * low-ALRI group = patients with <47 values of ALRI according to the median; ** low-SII group = patients with <580 values of SII according to the median.

**Table 4:** Best cut-off values for mortality and severity in COVID-19 patients.

	Mortality					Severity				
	Cut-Off	Sensitivity	Specificity	*p*-Value	AUC	Cut-Off	Sensitivity	Specificity	*p*-Value	AUC
FIB-4	2.19	70	64	<0.001	0.737	1.96	65	60	<0.001	0.684
APRI	0.47	65	62	<0.001	0.665	0.41	60	53	<0.001	0.597
NLR	3.65	65	61	<0.001	0.684	3.14	61	57	<0.001	0.623
ALRI	50.97	61	57	<0.001	0.619	43.54	60	49	<0.001	0.561
SII	617.61	63	55	<0.001	0.643	547.52	60	50	<0.001	0.588

## Data Availability

The data presented in this study are available on request from the corresponding author.
